# Emerging strategies for treating autoimmune disease with genetically modified dendritic cells

**DOI:** 10.1186/s12964-024-01641-7

**Published:** 2024-05-07

**Authors:** Yunhan Ma, Ruobing Shi, Fujun Li, Haocai Chang

**Affiliations:** 1 School of Medicine, Jiangsu University, Zhenjiang, 212000 China; 2 Ruikang Hospital Affiliated to Guangxi University of Chinese Medicine, Nanning, 530000 China; 3 MOE Key Laboratory of Laser Life Science, Institute of Laser Life Science, College of Biophotonics, South China Normal University, Guangzhou, 510631 China; 4 Guangdong Provincial Key Laboratory of Laser Life Science, College of Biophotonics, South China Normal University, Guangzhou, 510631 China

**Keywords:** Tolerogenic dendritic cells, Gene editing, Autoimmune disease, Organ transplantation

## Abstract

Gene editing of living cells has become a crucial tool in medical research, enabling scientists to address fundamental biological questions and develop novel strategies for disease treatment. This technology has particularly revolutionized adoptive transfer cell therapy products, leading to significant advancements in tumor treatment and offering promising outcomes in managing transplant rejection, autoimmune disorders, and inflammatory diseases. While recent clinical trials have demonstrated the safety of tolerogenic dendritic cell (TolDC) immunotherapy, concerns remain regarding its effectiveness. This review aims to discuss the application of gene editing techniques to enhance the tolerance function of dendritic cells (DCs), with a particular focus on preclinical strategies that are currently being investigated to optimize the tolerogenic phenotype and function of DCs. We explore potential approaches for in vitro generation of TolDCs and provide an overview of emerging strategies for modifying DCs. Additionally, we highlight the primary challenges hindering the clinical adoption of TolDC therapeutics and propose future research directions in this field.

## Background

**Fig. 1:**
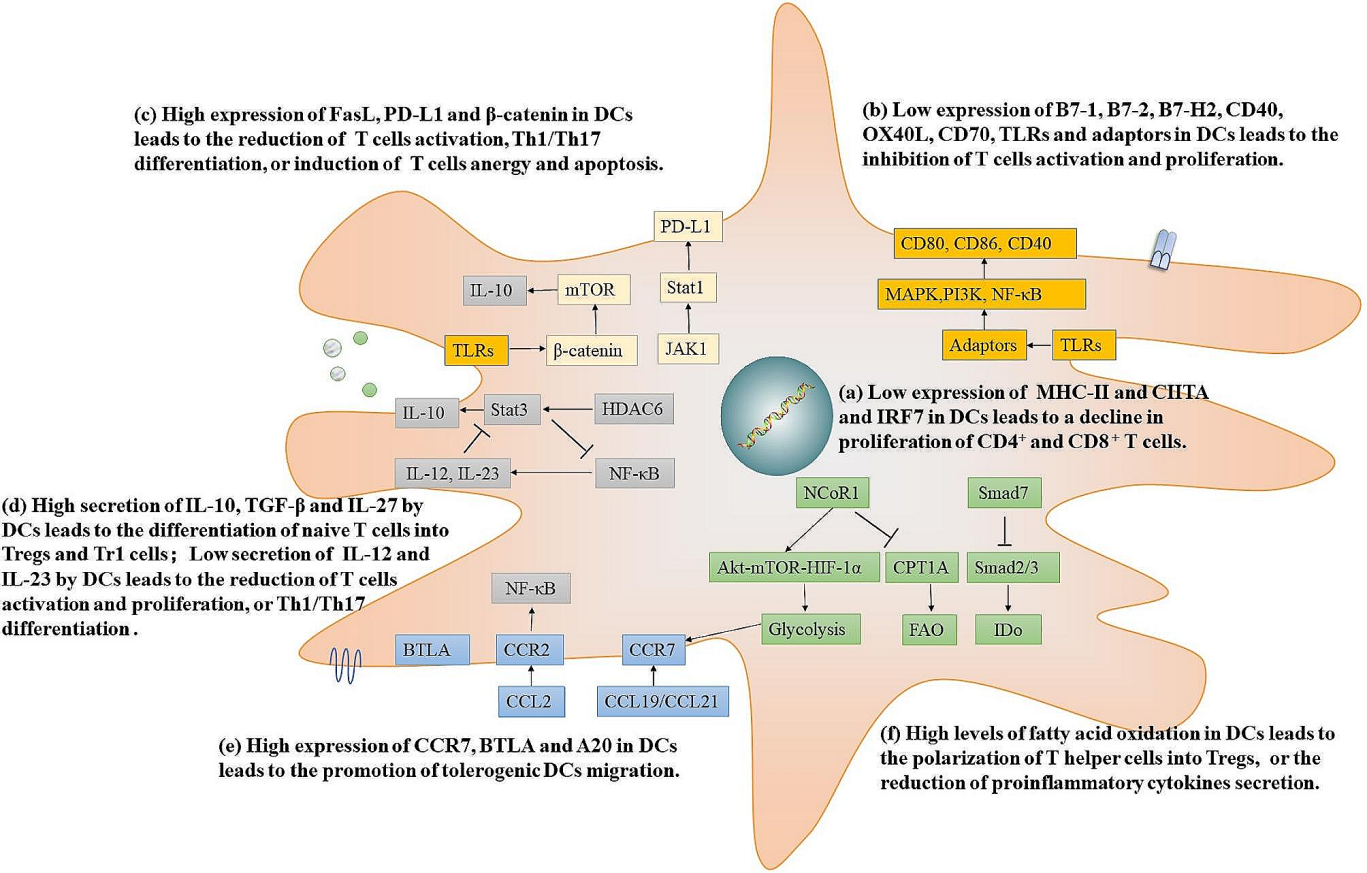
Recent strategies to modification of DCs to promote tolerogenic phenotype and function (**a**) Inhibiting the expression of MHC-II is associated with the downregulation of antigen peptide presentation and the recognition of T cells. (**b**) Decreasing the expression of co-stimulatory molecules, TNFSF, TLRs and their adaptors is associated with DC tolerogenic function. (**c**) Inducing the expression of FasL, PD-L1 and β-catenin promotes T cells apoptosis. (**d**) Overexpression of IL-10 and IL-27 in DCs exhibits a higher suppressive capacity. (**e**) Promoting the expression of CCR7 and BTLA imparts tolerogenic properties and aids in the maintenance of peripheral tolerance. (**f**) Upregulation of IDo and downregulation NCoR1 expression in DCs contribute to the polarization of Th cells into Tregs

The current standard treatment approach for transplant rejection and autoimmune diseases involves non-specific immunosuppressive medications. Despite the emergence of advanced antibody-based biologics as potential treatment options, the majority of rejection and autoimmune diseases still remain incurable. Furthermore, these medications inevitably carry the risk of serious immediate or delayed adverse reactions, such as life-threatening infections and cancer [1]. Addressing the root cause of autoimmunity, specifically the loss of tolerance to autoantigens, or preventing the induction or manifestation of undesirable immune responses in transplantation and allergy, represents a crucial progression in steering clear of general immunosuppression. Recent basic research findings have suggested that cell-based immunotherapy could offer a more effective and targeted approach to induce and establish long-term tolerance. The majority of ongoing research is centered around generating and therapeutically applying dendritic cells (DCs), aiming to restore tolerance in autoimmune diseases and prevent transplant rejection [2, 3]. Consider their significant role in maintaining peripheral tolerance in both mice and humans. Thus far, clinical trials have extensively investigated the safety, feasibility, and effectiveness of diverse tolerogenic DCs (TolDCs) in treating patients, showing no associated side effects and good tolerability [3, 4]. However, further approaches may be required to enhance the efficacy and specificity of TolDCs. This review explores the impact of gene modifications on the application of DC therapy (Fig. 1). We underline the regulatory mechanisms of targeted gene, and their potential use to improve the efficacy and patient survival in cell-based therapies, ultimately aiming to induce tolerance.

## The path to tolerogenesis in DCs

### Generation and characteristics of TolDCs

DCs with stable, semi-mature phenotypes and tolerant properties are often referred to as TolDCs, which play a critical role in preventing immune rejection and inflammation. In vitro, TolDCs are typically generated from human blood monocytes (CD14^+^) and rodent bone marrow precursors, or nonhuman primates CD34^+^ cells. The differentiation of these cells into immature DCs (iDCs) is facilitated by granulocyte-macrophage colony-stimulating factor (GM-CSF). Typically, cytokines (such as IL-10, TGF-β), and immunosuppressive drugs (such as dexamethasone, rapamycin, mycophenolic acid and vitamin D3) are added during the culture to enhance TolDCs tolerance [5]. TolDCs exhibit certain characteristics at the end of the culture period to be identified as such: (i) reduced or absent expression of cell surface markers, including major histocompatibility complex II (MHC-II), co-stimulatory molecules (CD80 and CD86), activation markers (CD40); (ii) resistance to maturation stimulation, as tested using pathogen and/or inflammatory signals, lipopolysaccharide (LPS), CD40 ligand, DC maturation cocktail, and TNF-α; (iii) minimal potential to induce allogeneic T cell proliferation upon stimulation, along with the ability to produce IL-10 and support the proliferation of regulatory T cells (Tregs) [6].

## Transcriptional determinants

In inflammatory environments, such as those found in the example, IFN-γ interacts with its receptor to activate the transcription factor signal transducer and activator of transcription 1 (Stat1). This activation facilitates the rapid development of iDCs into a fully functional mature phenotype [7]. Conversely, inhibition of IFN-γ receptor through gene editing techniques suppresses DC maturation [8]. While iDCs are typically associated with tolerance and mature DCs (mDCs) with an immunogenic phenotype, the relationship between maturation and the acquisition of an immunogenic phenotype is not always evident. In fact, studies have reported that in mice, iDCs can undergo stable maturation in vivo, leading to mDCs with tolerance characteristics [9]. Some studies have suggested that DCs can also develop with tolerogenic or immunogenic phenotypes even after they have matured [9].

The Wnt-β-catenin signaling pathway is crucial for immune homeostasis and tolerance in DCs [10]. Specifically, β-catenin expressed in DCs is essential for inducing Tregs and producing anti-inflammatory cytokines [11]. Mechanistically, β-catenin translocates from the cytoplasm to the nucleus and collaborates with members of the T cell factor/lymphoid enhancer factor family to regulate the transcription of tolerogenic genes [12]. While NF-κB signaling is necessary for the differentiation of immunogenic DCs, emerging research suggests that downstream transcription factors of NF-κB play a role in the regulation of immune homeostasis and tolerance in DCs [13]. Based on these findings, it can be inferred that transcription factors are crucial factors in determining the immunogenicity or tolerogenicity of DCs. Nonetheless, the specific processes that govern the differentiation of distinct DC subpopulations from their progenitor cells, as well as the underlying mechanisms driving the tolerogenic or immunogenic response, remain to be fully elucidated.

Transcription factors collaborate with epigenetic enzymes to target specific genomic regions, leading to epigenetic modifications. It is widely acknowledged that epigenetic marks can directly impact or influence the phenotype and function of cells. Multiple studies have demonstrated the involvement of various epigenetic mechanisms in the development of tolerance in DCs. For instance, prostaglandin E2 induces the upregulation of DNA methyltransferase DNMT3A, facilitating the hypermethylation and silencing of immunogenic genes. As a result, it promotes the tolerogenic function of human DCs to inhibit CD8^+^ T cell proliferation and IFN-γ production [14]. Specific protein palmitoylation is another process linked to the acquisition of tolerance in DCs. The zDHHC family of S-acylation enzymes, exemplified by zDHHC2, mediates endothelial dysfunction in the systemic inflammatory response syndrome [15]. zDHHC2 specifically regulates substrate protein and membrane binding, as well as cross-talk with phosphorylation [16]. Deficiency of zDHHC2 in human plasmacytoid DCs (pDCs) cellular model has been found to augment immune tolerance by inhibiting IFN-α production [17]. While the research on the potential contribution of histone modifications and DNA methylation to the acquisition of tolerance in DCs is limited, various studies provide support for the relevance of epigenetic modifications in this context. As an illustration, the in vitro differentiation of human monocytes into DCs is accompanied by Stat6-mediated acquisition of DNA methylation changes. These changes are crucial for establishing the distinct phenotype of DCs [18]. This finding confirms the role of DNA methylation in maintaining the stability of tolerogenic phenotypes.

### Metabolic determinants

In general, DCs have the capacity to induce self-immune tolerance through autocrine TGF-β signaling [19]. TGF-β induces the expression of indoleamine 2,3-dioxygenase (IDo), an enzyme that participates in the catabolism of tryptophan and serves an immunosuppressive function in DCs [20]. Furthermore, Smad7 acts as a potent negative regulator of TGF-β signaling by preventing the binding of Smad2/3 to the TGF-β receptor II, which is essential for transducing TGF-β signaling [21]. Smad7^−/−^ DCs exhibit elevated levels of IDo expression. Mice devoid of Smad7 specifically in DCs are resistant to the development of encephalomyelitis (EAE). This resistance is attributed to an enhanced presence of Tregs in the central nervous system and a diminished number of effector T cells in the brain [22].

Nuclear receptor corepressor 1 (NCoR1) is considered to play a crucial role in maintaining homeostasis and regulating metabolism in the body [23]. Recent research has indicated that the deficiency of NCoR1 induces a robust tolerogenic response in DCs. In a model of parasitic infection, the adoptive transfer of NCoR1-deficient DCs leads to the polarization of T helper (Th) cells into Tregs in vivo, resulting in an augmentation of the disease phenotype [24]. Mechanistically, NCoR1 deficiency drives Akt-mTOR-HIF-1α axis-mediated glycolysis and CPT1A-driven beta-oxidation [25]. Thioredoxin-binding protein-2 (TBP-2) plays a key regulator role in maintaining cellular redox balance. The TBP-2^−/−^ DCs exhibited comparable levels of MHC-II and co-stimulatory molecules upon LPS stimulation compared to wild-type DCs. However, there was a reduction in the production of IL-12p40, IL-12p70, and IL-6 [26].

### Crosstalk with other immune cells

An important characteristic of DCs and natural killer (NK) cells is their ability to regulate each other through intercellular contact, leading to Th1 polarization and the secretion of cytokines [27]. NK cells induced DCs maturation and secretion of IL-12p70 [28]. Conversely, mDCs exert cytotoxic functions by activating NK cells. Galectin-3 (Gal-3), an endogenous lectin, has been found to have both promoting and anti-inflammatory effects in various disease conditions. It has been reported that the deletion of Gal-3 in DCs inhibits NK cells from producing pro-inflammatory cytokines [29]. However, some studies suggest that effective interaction between DCs and NK cells can only occur through direct cell-cell contact [30], which is facilitated by interactions between tumor necrosis factor superfamily (TNFSF) ligands and their homologous receptors. For example, the binding of the transmembrane protein tmTNF expressed by DCs and tmTNFR2 expressed by NK cells induces the secretion of IL-12p70 by DCs and TNF-γ by NK cells [31]. This implies that secreted pro-inflammatory cytokines may be involved in the subsequent cell contact.

TolDCs have the ability to promote peripheral tolerance by facilitating the differentiation of Tregs. In asthma models, the Tregs induced by TolDCs displayed greater regulatory activity compared to natural Tregs with the same T-cell receptor (TCR) specificity [32]. During homeostasis, DCs also present self-antigens or harmless antigens, thus promoting the differentiation of Tregs [33]. Interestingly, these DCs exhibit a mature phenotype characterized by high expression levels of MHC-II and co-stimulatory molecules, yet they do not induce an immune response [34].

Co-stimulatory signals, such as CD80/CD86-CD28 [35] and CD40-CD154 [36] between DCs and T cells contribute to the production of Tregs. Mice devoid of CD80 or CD86 specifically in DCs showed a decrease in the number of peripheral Tregs without over-activation of T cells [34]. DCs expressing the transcription factor IRF4 have been found to exhibit a mature phenotype during homeostasis and promote the production of Tregs by enhancing the expression of genes required for antigen presentation and T-cell tolerance, such as retinaldehyde dehydrogenase and PD-L2 [37]. Conversely, a decrease in the expression of IRF4 promotes the response of DCs to oxidative stress and inflammatory cytokines [38]. Lastly, TolDCs have the ability to directly eliminate T cells through clonal clearance. For instance, DCs express the TNF-related apoptosis-inducing ligand (TRAIL), which interacts with death receptors in T cells, thereby promoting T cell apoptosis by activating the caspase pathway [39].

However, the treatment of TolDCs in autoimmunity and transplant rejection faces two major problems: The first problem is the inflammatory environment within the body. In such an in vivo inflammatory environment, TolDCs are prone to developing into mDCs and even immunogenic DCs due to their instability. To overcome this challenge, semi-mature TolDCs, which exhibit a more stable phenotype and are less prone to differentiation, have shown the ability to prolong graft survival, particularly under inflammatory stimulation compared to classical TolDCs. Common approaches for their generation include treatment with drug cocktails or immunomodulatory cytokines and exposure to apoptotic cells [40]. Another problem to consider is the adjunctive use of immunosuppressants. Clinical studies have consistently shown that adoptive transfer therapy using TolDCs still requires the synergistic effects of immunosuppressants. In vitro experiments conducted on mouse and human DCs have demonstrated that immunosuppressant-treated DCs exhibit enhanced tolerance functions. For example, rapamycin treatment assists TolDCs in inducing increased expression of immunoglobulin-like transcript 3 and 4 (ILT3 and 4), thereby contributing to the effective prolongation of graft survival. Conversely, cyclosporine-treated DCs have exhibited impaired migratory function [41]. Nonetheless, it is important to note that these strategies do not completely eliminate the generation of immunogenic DCs. Therefore, further improvement in the tolerance characteristics and stability of DCs can be achieved by modifying the DC genome.

### Inhibiting the expression of MHC-II

TolDCs express low level of MHC-II, which is essential for the presentation of antigen peptides for recognition by T cells. Consequently, suppressing the expression of MHC-II in DCs through gene editing techniques exhibits several TolDC-like characteristics, including immunosuppressive ability and the ability to maintain a stable phenotype even under inflammatory conditions in vivo [42].

**Fig. 2:**
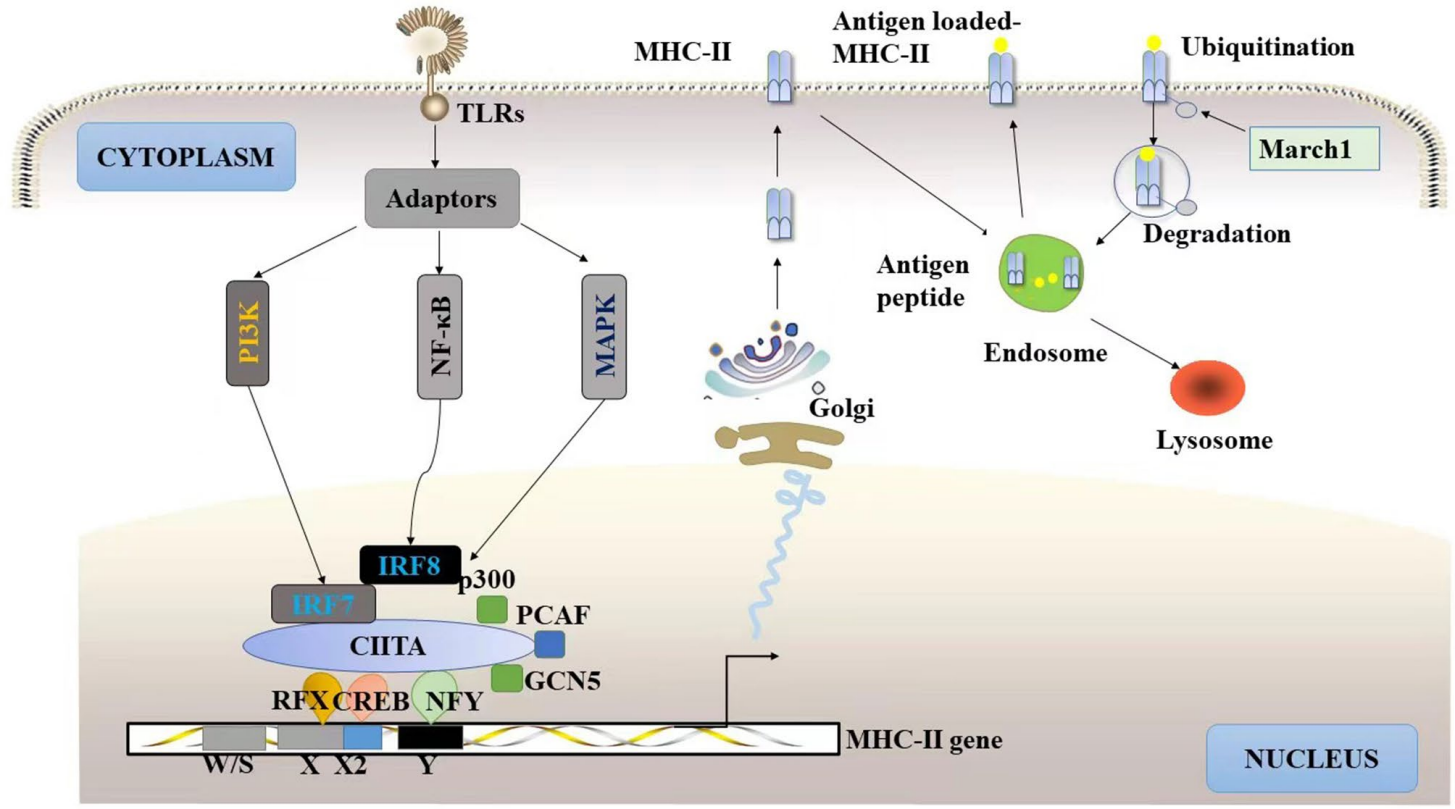
Model for MHC-II trafficking TLRs stimulate immunogenic DC development by inducing MHC-II synthesis *via* activation of the NF-κB and PI3K signaling pathway. The recruitment of the CIITA to the enhancer is necessary for MHC-II transcription, CIITA interacts with various members of the RFX, CREB and NF-Y complexes to positively regulate MHC-II gene transcription. CIITA recruits CBP/p300, PCAF, and GCN5 to the promoter, enhancing MHC-II transcriptional activity. Newly synthesized MHC-II proteins are transported *via* the Golgi to the endosome. Here, antigen peptide is loaded onto MHC-II, then antigen loaded-MHC-II traffic to the plasma membrane *via* transport vesicles for antigen presentation. In resting DCs, MHC-II ubiquitination enhances the kinetics of degradation of peptide-bound MHC-II molecules and prevents recycling of internalized molecules back to the cytoplasm. Additionally, March family has been shown to be involved in MHC-II ubiquitination

Sequence comparisons of human and mouse MHC-II genes have revealed the presence of short cis-acting sequences upstream of their transcription start sites, named W/S/Z, X1, X2, and Y box (SXY modules). The RFX, NF-Y and cAMP response element binding protein (CREB) are synergically bound to the SXY modules to form a stable nucleoprotein complex called the MHC-II booster, in which the RFX complex is a key component in the assembly of the element [43]. Enhancer formation and the recruitment of the non-DNA-binding transcription factor-MHC-II transactivator (CIITA) to the enhancer are necessary for MHC-II transcription [44] (Fig. 2). CIITA plays a crucial role in recruiting histone acetyltransferases such as CBP/p300, PCAF, and GCN5 to the MHC-II promoter, leading to the activation of acetylation markers [45]. Importantly, CIITA itself possesses histone acetyltransferase activity [46]. Interestingly, histone hyperacetylation not only inhibits the recruitment of histone deacetylase-induced enhancer components but also induces MHC-II expression in the absence of CIITA [47]. These findings suggest that histone acetylation serves multiple functions on the MHC-II promoter, modifying CIITA and chromatin structure to promote more efficient transcription initiation [48].

Downstream signaling intermediates play a vital role in regulating the expression of key transcription factors necessary for DC differentiation. Deletion of transcription factors such as IRF8, IRF4, or PU.1 leads to the loss of specific DC subpopulations and results in reduced MHC-II expression on bone marrow-derived DCs (BMDCs) in response to GM-CSF stimulation [49]. In addition, the Src-like adaptor protein (SLAP) functions as a negative regulator of the GM-CSF receptor. However, SLAP/SLAP2^−/−^ BMDCs exhibit decreased levels of MHC-II expression. This suggests that the mechanism behind this phenomenon may be linked to the excessive activity of the GM-CSF signaling pathway, which ultimately impacts MHC-II transcription [50].

The increased expression of MHC-II on the surface of DCs is attributed to a reduction in the ubiquitination level of lysine residues located in the cytoplasmic tail of the MHC-II-β chain. This results in a decreased rate of endocytosis and degradation of MHC-II. The process is tightly regulated by the E3 ubiquitin ligase known as membrane-associated ring-CH-type finger 1 (March1). Deletion of March1 or the mutation of the β-tail lysine residue (K225R-DCs) in MHC-II leads to a decline in MHC-II ubiquitination within the cytoplasmic recycling pathway. Subsequently, this decrease contributes to an enhanced expression of MHC-II on the surface of DCs. Although the phagocytosis of antigens by K225R-DCs increases, their ability to present antigens to T cells is diminished. Additionally, single-cell sequencing analysis confirmed that genes related to K225R-DCs are highly expressed, specifically in down-regulated cellular activation pathways [51]. Ubiquitination also plays a role in regulating MHC-II transcription levels. Another E3 ubiquitin ligase, HMG-CoA reductase degradation protein (Hrd1), promotes the degradation of B lymphocyte-induced maturation protein-1 in DCs through ubiquitination, thereby enhancing MHC-II transcription. Hrd1^−/−^ BMDCs exhibit decreased expression of MHC-II, which is associated with a delay in the onset of EAE in DC-specific Hrd1^−/−^ mice [52].

### Immunomodulatory ligands and receptors involved in TolDCs

#### The B7 family

The B7 family of co-stimulatory molecules is known to play a pivotal role in T cell activation. Among these, B7-1 (CD80), B7-2 (CD86), B7-H2 (ICOSL), and B7-H7 (HHLA2) provide activation signals to T cells, while B7-DC (PD-L2), B7-H1 (PD-L1), B7-H3 (CD276), B7-H4 (B7S1/B7x/Vtcn1), B7-H5 (VISTA/GI24/Dies/PD-1 H), and BTNL2 inhibit T cell activation (Table 1). Several immunotolerance strategies for inducing TolDCs, including deletion of CD80 [53], CD86 [54] or ICOSL [55], and overexpression of PD-L1 [56] have been shown to be effective in inducing transplantation tolerance and inhibiting autoimmune diseases. Machen et al. engineered an oligodeoxynucleotide antisense (AS-ODN) that targets the transcription of CD80 and CD86 in BMDCs, leading to the inhibition of NO, TNF-α, and IL-12 secretion [57] and a delayed onset of type 1 diabetes (T1D) in NOD mice. These promising preclinical results pave the way for clinical trials involving AS-ODN-treated human monocyte-derived DCs [58].

**Table 1:** Members of B7 family

Ligand	Express on immune cells	Receptor	Function	Signaling pathway on T cells	Related autoimmune disease
B7-1 (CD80) B7-2 (CD86)	DCs, Langerhans’ cells, activated macrophages and B cells.	CD28 PD-L1	Inducing the activation and proliferation of naive T cells and IL-2 production.	Activating PI3K-Akt, JNK, ERK, signaling pathways and inducing NF-κB, NFAT, mTOR, GLUT1 [104].	Belatacept and abatacept, high affinity CTLA4-Ig that blocks CD28-CD80/CD86 interactions, have been approved for the prevention of rheumatoid arthritis [105] and acute rejection in adult kidney transplantation [106].
		CTLA-4	Inhibiting T cell responses, IL-2 production and Th2/Th17 differentiation. Inducing Tregs generation.	Inhibiting cell cycle and NFAT nuclear translocation. Inducing GIT2-αPIX-PAK- PKC-η axis [107].	
B7-DC (PD-L2) B7-H1 (PD-L1)	DCs, monocytes, macrophages, B cells and activated T cells.	PD-1	Inhibiting T cell proliferation and cytokines (IL-10, IFN-γ) production. Inducing Tregs generation.	Inhibiting the PI3K-Akt, MEK-ERK signaling pathways and cell cycle. Inducing CTLs apoptosis.	The PD-L1 inhibitor atilizumab and dvizumab have been approved for the prevention of acute rejection in liver transplantation [108].
B7-H2 (ICOSL)	DCs, B cells and macrophages.	ICOS	Inducing the activation and proliferation of T cells, T cell-dependent B cell activation, Th2 differentiation and IL-4 production.	Activating PI3K-Akt signaling pathway.	ICOS-ICOSL blocking agents lack efficacy in prolong kidney allograft survival in a NHP model [109].
B7-H3 (CD276)	T cells, CD11c^+ ^DCs, NK cells and macrophages.	Unidentified	Predominantly inhibiting T cell proliferation and Th1 response.	Inhibiting NFAT, NF-κB and AP-1 expression [110].	B7-H3 antibody results in earlier onset and worse disease in mouse encephalomyelitis model [111]. B7-H3 expression negatively regulates acute graft-versus-host disease in mice [112].
B7-H4 (B7S1/B7x/Vtcn1)	CD11c^+^ DCs, BMDCs, peritoneal macrophages and splenic B cells.	Unidentified	Inhibiting T cells proliferation and cytokines production. Promotes Tregs development.	Arresting cell cycle [113].	B7-H4 mAb enhances mouse encephalomyelitis severity [114]. Treatment of NOD mice with B7-H4-Ig ameliorates the incidence of diabetes [115]. B7-H4 promotes the viability of islet grafts [116].
B7-H5 (VISTA/GI24/Dies/PD-1 H)	Macrophages, mature BMDCs, neutrophils and CD11c^+^ DCs, and to a less extent on T cells and activated Tregs.	Unidentified	Inhibiting T cells proliferation and cytokines production. Supporting Tregs survival.	Arresting cell cycle [117]. Inhibiting SLP76, PLC-γ1, Akt, and ERK1/2 expression [118].	B7-H5 agonist alleviates autoimmune lupus. B7-H5 agonists or mAb prevents graft-versus-host disease [119], and prolongs corneal allograft survival in mouse [120].
B7-H7 (HHLA2)	Monocytes and induced on B cells.	TMIGD2	Inhibiting T cells proliferation, cytokines (IFN-γ, TNF-α, IL-5, IL-10, IL-13, IL-17 A, and IL-22) production.	Inhibiting ERK1/2, Akt and NF-κB signaling pathways by aggregating SHP1/2 [121].	Predominantly focus on oncology.
BTNL2	DCs	Unidentified	Inhibiting the activation and proliferation of T cells, cytokines production, Th differentiation. Inducing Tregs generation.	Inhibiting Akt signaling pathways. Inducing Foxo1 expression [122].	Treatment of recombinant BTNL2-IgG2a Fc fusion protein ameliorates type 1 diabetes [123] and graft-versus-host disease in mice [124].

The expression of costimulatory molecules on the surface of DCs is regulated by multiple mechanisms. The transcription factor Stat1 plays a critical role in acquiring tolerance characteristics, specifically through JAK-Stat1 signaling that promotes PD-L1 expression [59]. In a mouse model with DCs lacking JAK1 (JAK1^−/−^ DCs), PD-L1 expression was reduced, leading to increased activation and proliferation of T cells and a poor prognosis in EAE [59]. These findings suggest that targeting JAK in DCs could be beneficial for treating autoimmune diseases [60]. Notably, the absence of JAK1 did not impact the expression of other inhibitory molecules, such as CD270, PD-L2, and galectin-9 [61].

### The TNF ligand and TNF receptor superfamilies (TNFRSFs)

The immunoregulatory function of several TNFSF members in DCs has been well established (Table 2). These include OX40 ligand, TNFRSF5 (CD40), TNFSF15 (TL1A), CD70, 4-1BB ligand, and TNFRSF18 (GITR). In an inflammatory environment, OX40L is primarily expressed on antigen-presenting cells (APCs) and binds to the transmembrane glycoprotein OX40 on T cell surface, leading to the activation of NF-κB and NFAT signaling pathways. This activation promotes sustained activation and expansion of effector T cells and memory T cells [62]. By blocking OX40L-OX40 signaling, the differentiation of Tregs can be induced, thereby preventing autoimmune reactions [63]. The interaction between CD40 and CD154 (CD40 ligand) plays a central role in immunology, with diverse effects on T cell activation. Consequently, conditional knockout of CD40 in DCs leads to reduced Th1 immune responses and decreased plasma IFN-γ levels in DC-specific CD40^−/−^ mice, concomitant with a reduction in the occurrence of atherosclerosis in this mouse model [64]. Besides the classical T cell activation pathway mediated by CD80/CD86-CD28 signaling, the CD70-CD27 axis represents an equally crucial co-stimulatory signaling pathway for T cell activation [65]. Clinical trials have revealed that the silencing of CD70 in DCs can reduce the polarization of Th1 and Th2 cells, suggesting its potential feasibility for treating immune thrombocytopenia [66].

**Table 2:** Members of tumor necrosis factor superfamilies (TNFRSF)

Ligand	Express on immune cells	Receptor	Effects	Mechanism on T cells	Related autoimmune disease
4-1BB ligand	DCs, macrophages and activated B cells.	4-1BB	Inducing CTL responses.	Induction of the NF-κB, c-Jun and p38 downstream pathways [125].	Blockade of 4-1BBL-4-1BB interaction prevents acute transplant rejection [126, 127] and experimental autoimmune myocarditis [128] in preclinical trials.
TNFRSF5 (CD40)	Macrophages and DCs.	CD154	Inducing Th1 responses and IFN-γ production.	Induction of the ERK1/2, JNK, PAK and PI3K-Akt signaling pathways [129], and inhibiting apoptosis [130].	Anti-CD40 mAb is being used in clinical trials to treat rheumatoid arthritis [131] and pig organ xenotransplantation [132].
TNFSF15 (TL1A)	DCs and macrophages.	DR3	Inducing effector/memory CD4^+^ T cell proliferation and cytokine (IFN-γ, TNF-α, IL-1α) production. Inhibitings Treg differentiation.	Induction of the MAPK, NF-κB and PI3K-Akt signaling pathways [133].	TL1A is specifically elevated in the blood and synovial fluid of patients with rheumatoid arthritis and systemic sclerosis. TL1A blockade could be a potential therapeutic strategy in autoimmune disease [134–136].
CD70	DCs and B cells.	CD27	Inducing the activation and proliferation of T cells, cytokine (IL-2, IL-4, IL-5, IL-6, IL-12, IFN-γ) production and antibody secretion.	Induction of the NF-κB, JNK, Ap-1, ERK and MAPK signaling pathways [137].	Anti-CD70 mAb inhibits encephalomyelitis and cardiac allograft rejection in mice [138, 139].
GITRL	Macrophages, DCs and B cells.	GITR	Inducing the activation and proliferation of T cells and pro-inflammatory cytokine production.	Inhibiting apoptosis. Induction of the MAPK, NF-κB signaling pathways [140–142].	Blockade of GITRL-GITR interaction prevents encephalomyelitis [143], allergic asthma [144] and experimental colitis [145] in mice.
OX40 ligand	DCs, B cells, and macrophages.	OX40	Inducing the activation and proliferation of T cells, memory T cells formation and CTL responses. Inhibiting Treg differentiation.	Inhibiting apoptosis. Induction of the NF-κB, MAPK, NFATc signaling pathways [146].	OX40L-blocking antibody KY1005 ameliorates graft-versus-host disease in nonhuman primate [147]. KY1005 has completed phase 2a clinical trial in treat atopic dermatitis [148].

### The TLRs family

TLRs are well-defined microbial pattern recognition receptors expressed on APCs. They recognize pathogen-associated molecular patterns, leading to the activation of immediate inflammatory responses and subsequent intracellular signal transduction pathways. The cytoplasmic domain of TLRs recruits various adaptors that activate specific downstream signaling pathways, initiating innate immune responses and inducing the secretion of pro-inflammatory cytokines [67]. The selection of adaptors determines the specificity of TLR signaling, resulting in the activation of specific TLR-defined transcription factors such as NF-κB, PI3K, mitogen-activated protein kinases (MAPK), c-Jun, and IRF-3 (among others) [67–69]. For instance, myeloid differentiation factor 88 (MyD88) serves as the adapter protein for TLR2 and TLR4 [70], activating the NF-κB and MAPK signaling pathways [69]. TLR2/4^−/−^ DCs exhibit decreased expression of CD86, CD80, and CD40 [71], while transplantation of corneas into TLR^−/−^ recipients leads to prolonged graft survival [71].

### Other co-inhibitory molecules on DCs

The expression of co-inhibitory molecules on TolDCs triggers contact-dependent inhibitory signals in T cells, suppressing proliferation and promoting clonal deletion (Table 3). One such example is the ILT family, which acts as inhibitory surface receptors structurally and functionally related to killer inhibitory receptors [72]. The specific ligand/receptor pathways of ILT are still limited, but many TolDCs express ILT, which subsequently promotes tolerance by inhibiting T cell proliferation [73]. Furthermore, iDCs express Fas ligand (FasL), which binds to Fas molecules on T cells, leading to T cell apoptosis [74]. Consequently, the adoptive transfer of FasL-overexpressing human iDCs has been shown to prolong the survival of liver allografts in rat recipients [75]. Additionally, inducing the generation of DCs through embryonic stem cells could represent a cost-effective approach for developing therapeutic vaccines that regulate immune tolerance. In an EAE mouse model, the adoptive transfer of TRAIL-overexpressing embryonic stem cell-derived DCs promotes the generation of Tregs and limits disease development [76].

**Table 3:** Members of other co-inhibitory molecules on DCs

Co-inhibitory molecules	Ligand/Receptor	Effects	Related transplantation and autoimmune disease
ILT2/3/4	HLA-G	Inhibiting the function of effector T and NK cells, DCs maturation. Inducing Tr1 cells differentiation [149]. Inducing Tregs differentiation.	Treatment of recombinant human ILT3.Fc protein ameliorates encephalomyelitis in mice [150]. ILT-HLA-G interaction prevents transplant rejection graft-versus-host disease in mice [151, 152].
FasL	Fas	Inducing T cells apoptosis.	FasL microgels induce immune acceptance of islet allografts in nonhuman primates [153].
TIM-3	Gal-9	Inducing Th1 cells deletion. Inhibits generation of IFN-γ in CD8^+^T cells [154, 155].	Anti-TIM-3 treatment aggravates lung inflammation and fibrosis in mice [156]. TIM-3-overexpressing DCs ameliorates allograft rejection in mouse composite tissue allotransplantation [157].
TRAIL	DR3/4/5	Inducing T cells apoptosis [158].	Treatment of recombinant TRAIL ameliorates experimental colitis [159], arthritis [160] in mice. Blockade of TRAIL-DR3 interaction prevents acute rejection in mouse kidney transplantation [161].
BTLA	HVEM	Inhibiting DCs maturation [94], proinflammatory cytokines production in DCs [162]. Inducing Tregs differentiation [163].	Overexpression of BTLA ameliorates allograft rejection in rat kidney allotransplantation [164, 165].

### Regulating the secretion of cytokines

#### Inducing the secretion of anti-inflammatory cytokines

Anti-inflammatory cytokines, such as IL-10 and TGF-β produced by TolDCs, induce the differentiation of naive T cells into Tregs and Tr1 cells [77]. The adoptive transfer of IL-10-overexpressing human iDCs to recipient rats has been shown to induce long-term survival of liver grafts [75]. Among them, Histone deacetylases (HDACs) regulate IL-10 transcription by impacting the phosphorylation of Stat3, a transcriptional activator of IL-10. Hence, overexpression of HDAC6 in DCs can increase IL-10 generation [78].

**Fig. 3:**
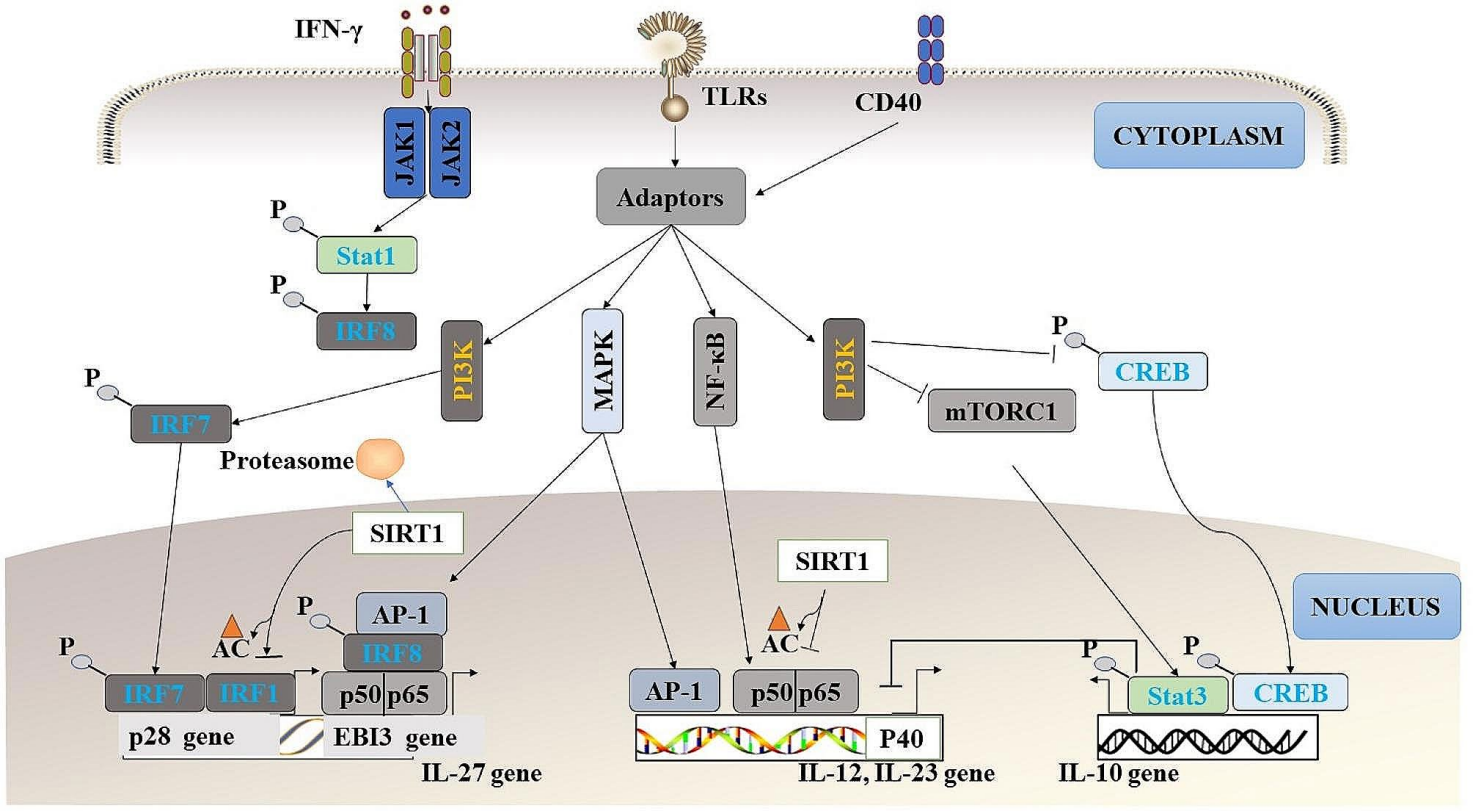
Generation of cytokines and their downstream signaling pathway in DCs. TLRs, CD40 and IFN-γ promote activation of intracellular signaling cascades, resulting in activation of transcription factors such as IRF7, NF-κB and AP-1. These regulate cytokine genes transcription, including IL-27, IL-12, IL-23 and IL-10. The expression of the IL27EBI3 subunit can be induced through intracellular signaling cascades coming from the TLR2/TLR4/TLR9/-adaptor MyD88-NF-κB signaling pathway. Other signaling pathways that participate in the induction of IL-27p28 synthesis are TLR4-adaptor MyD88-AP-1, TLR3/TLR4-adaptor TRIF-IRF3/IRF7 and IFN-γ-adaptor MyD88-Stat1-IRF8. The expression of the IL-12 and IL-23 can be induced through signals coming from the MAPK, JAK, and PI3K signaling pathway. SIRT1 is a deacetylase that inhibits gene transcription by removing acetyl groups from transcription factors. SIRT1 deacetylates the transcription factor IRF1, which drives the generation of IL-27. Additionally, SIRT1 limits the production of IL-12 and IL-23 by deacetylating p53 and p65 (also known as RelA), a subunit of NF-κB. Inflammation increases the nucleoplasmic transporting of SIRT1 to the cytoplasm. This will decrease SIRT1 protein levels through proteasomal degradation in the cytoplasm. At the same time, the transcription of NF-κB-dependent pro-inflammatory gene, such IL-12, is increased once SIRT1 is reduced. The expression of IL-10 through PI3K-mTORC1-Stat3 signaling pathway is inhibited in response to TLR stimulation. Ac: acetylation, P: phosphorylation

IL-27 is a heterodimeric cytokine composed of Epstein-Barr virus-induced gene 3 (EBI3) and p28 proteins, both of which are expressed by APCs, particularly DCs, in response to TLR stimulation [79] (Fig. 3). IL-27 limits the differentiation of CD4^+^ T cells into Th1, Th2, and Th17 cells while promoting their differentiation into IL-10-producing Tr1 cells [80]. Sirtuins 1 (SIRT1), a class III histone/protein deacetylase, serves as a negative regulator of IL-27 expression (Fig. 3). Mechanistically, SIRT1 deacetylates IRF1 to inhibit IRF1 binding to the promoter of IL-27, and deletion of SIRT1 in DCs abolish IL-27 expression and alleviates the occurrence of EAE in mice [81].

### Inhibiting the secretion of pro-inflammatory cytokines

NF-κB activation serves as a major inducer of pro-inflammatory cytokines production in DCs, triggered by various danger signals (Fig. 3). This activation leads to the secretion of inflammatory cytokines including TNF-α, IL-6, IL-8, IL-12, and IL-23, which play vital roles in inducing Th1- and Th17-mediated immune responses [82]. In multiple studies, the deletion of IL-23p19, and IL-12p40 in DCs has been shown to result in a tolerant phenotype [83]. The transcription of IL-12 and IL-23 is negatively regulated by Stat3, which hinders the recruitment of NF-κB to the p40 promoter, a shared subunit between IL-12 and IL-23 (Fig. 3). Additionally, Stat3 inhibits the role of positive transcription elongation factor b (P-TEFb) in IL-12p35 initiation transcription [84]. Overexpression of constitutively active Stat3 or a constitutive NF-κB repressor in DCs inhibits the secretion of IL-23 and IL-12, thereby alleviating the development of rheumatoid arthritis [85].

### Regulating the migration of DCs

The normal expression of chemokine receptors can provide some degree of evidence regarding the ability of DCs to effectively circulate and localize to the lymph nodes. In line with the role of mature molecules in initiating protective immunity and maintaining immune tolerance, impaired migration of TolDCs can lead to abnormal inflammatory activation, while excessive migration of immunogenic DCs can also result in abnormal inflammatory activation.

### Inhibiting the migration of immunogenic DCs

Preventing the migration of immunogenic DCs from bone marrow to secondary lymphatic organs has emerged as an effective strategy for preventing autoimmune reactions. Normally, the migration of immunogenic DCs from the bone marrow to the lymphoid organs is tightly regulated and guided by chemokines and their corresponding receptors expressed on DCs. Upon exposure to inflammatory and pathogenic signals, iDCs undergo maturation and upregulate the expression of chemokine receptor CCR7. CCR7 interacts with its ligands CCL19 and CCL21, leading to the migration of immunogenic DCs along the CCL21 gradient into the subcapsular sinuses of the lymph nodes and eventually reaching the T-cell-rich regions. In these regions, immunogenic DCs can induce the polarization of naive T cells [86].

Targeting CCR7 and CCR2 has been shown to decrease the migration of DCs as well as reduce the production of IL-12p70 [87]. Previous study demonstrated that CCR2 can influence the transcriptional activity of NF-κB, which in turn promotes the production of pro-inflammatory cytokines [88]. Interestingly, CCR7^−/−^ mice exhibited an autoimmune phenotype characterized by extensive lymphocyte infiltration in organs and an abundance of autoantibodies in the blood [89]. This phenotype could be attributed to the higher expression of CD80 and CD86 on CCR7^−/− ^DCs [90], suggesting a dual role of CCR7 in both immune response and immune tolerance.

Gal-1, highly expressed by human lymphoendothelial cells, selectively modulates the migration of specific DC subpopulations. It inhibits the migration of immunogenic DCs but has no effect on the migration of tolerogenic DCs. This differential migration is mediated by the difference in the 2o glycosylation of CD43, a Gal-1 receptor, between immunogenic DCs and tolerogenic DCs. Binding of Gal-1 to immunogenic DCs decreases the phosphorylation and activity of the protein tyrosine kinase Pyk2, thereby reducing their migration [91]. Furthermore, Gal-3 is concentrated in membrane ruffles in DCs exposed to chemokines, and its deficiency results in structural differences in membrane ruffles, suggesting that the absence of this protein in membrane microdomains may impair DC migration [92]. Deletion of Gal-1 in mice increases their susceptibility to autoimmune diseases [93], providing an additional mechanism for treating autoimmune diseases and transplant rejection.

### Inducing the migration of TolDCs

In homeostasis, iDCs undergo spontaneous maturation and upregulate the expression of CCR7, they then migrate to lymph nodes to present self-antigens or harmless antigens, thereby preventing immune reactions against these antigens. Overexpression of CCR7 in iDCs has been shown to increase their migration with immunotolerant phenotype [94]. A study has demonstrated that the concurrent expression of BTLA and A20 in DCs promotes the expression of CCR7, and confers tolerogenic properties by decreasing surface expression of CD40 and CD86 [95]. The chemokine receptor 6 (D6), a member of the “clearance receptor” family, has been found to eliminate more than 12 chemokines, including CCR1-CCR5 [96]. D6^−/−^ BMDCs exhibited decreased expression of MHC-II, CD40, CD80, and CD86 in response to LPS. Consequently, D6^−/−^ mice exhibited inhibited rejection of transplanted corneas [97] and delayed EAE occurrence [98].

### Optimizing TolDC therapy and future perspectives

Optimizing antigen specificity is a key factor in enhancing the therapeutic efficacy of TolDC products (Fig. 1). Directed DNA gene editing technologies, such as TALEN, ZFN, and CRISPR, have emerged as valuable tools for gene deletion. Although the application of these gene editing techniques in human DCs is still in nascent stages, there is a high likelihood of employing these technologies to generate more efficacious TolDC products. TLRs are prime examples, and these molecules have been demonstrated to be a co-stimulatory molecule for CTL response, which can be deleted using these approaches. Indeed, the benefits of CRISPR-mediated deletion of TLRs and its constraining effect on T cell responses have been validated in response to LPS stimulation, and clinical trials are currently investigating the potential advantages of this approach [99]. Another advantage of utilizing directed DNA gene editing technologies is that the directed gene deletion system is co-delivered with a DNA template to facilitate homologous recombination, they can be employed to insert genes of interest into specified genomic loci. This eliminates safety concerns associated with conventional viral methods and enables the modulation of ectopic gene expression using endogenous promoters and enhancers. For instance, melanoma manipulates the metabolism of DCs through the paracrine β-catenin pathway to induce local immune tolerance [100]. Utilizing CRISPR to insert β-catenin into the target gene locus, thus establishing an endogenous immune-privileged site capable of expressing IDo in a more physiologically relevant manner [100]. This concept is also of significant relevance for the future development of next-generation TolDC vaccines.

Current strategies for TolDC cell therapy utilize autologous cells, which are personalized and difficult to standardize, leading to high costs. Moreover, many autoimmune diseases stem from defective DCs. Consequently, using autologous cells for DC products often yields suboptimal results. For instance, vitamin D-TolDCs produced by multiple sclerosis patients display transcriptomic differences compared to those of healthy individuals [101]. Using gene-editing technology to repair defective DCs before reinfusion is an imaginative solution. Allorecognition primarily arises from the interaction of human leukocyte antigen (HLA) molecules between the donor and recipient. The most effective approach to maintaining autoimmune hyporeactivity and inducing long-term allograft survival involves eliminating HLA mismatch molecules. DCs differentiated from HLA-DR-knockout induced pluripotent stem cells have demonstrated immune tolerogenicity and overcame the limitations associated with cell origin [102]. Unfortunately, this approach also renders the cells susceptible to NK cell-mediated killing, a consequence of “loss of self”. Furthermore, DCs derived from different donors exhibit distinct responses to the same stimulus, such as LPS, suggesting the combined influence of genetic factors and environmental exposure in determining DC functionality and size [99]. Therefore, it is crucial to comprehensively characterize the inflammatory, transcriptomic, epigenomic, and metabolic profiles of each disease and conduct in-depth phenotypic analysis to discern the disparities between monocytes and TolDCs generated from healthy individuals versus those with autoimmune diseases. Such efforts are essential in preparing TolDC products with well-defined immunomodulatory capabilities.

## Conclusions

In recent years, clinical trials have provided evidence of the safety of TolDC treatment, marking the emergence of a new era in cell-based immunotherapy for autoimmunity and transplant rejection. However, in order for TolDC therapy to become a preferred treatment option, strategies to enhance the potency of these cells need to be explored. Currently, it remains unclear whether TolDCs constitute a distinct lineage or merely represents a specific activation state of conventional DCs. It is conceivable that the tolerogenic phenotype is governed by specific signaling pathways and transcriptional programs, such as those regulated by Stat3, AhR, and Socs2 [103]. Thus, understanding the pathways through which TolDCs develop tolerance mechanisms at the transcriptome, metabolome, and epigenome levels is crucial. Preclinical studies have demonstrated that immune tolerance and antigen specificity can be conferred to TolDCs using gene editing techniques. Thus, gene-editing techniques are needed to be developed to ensure the accuracy of editing. Consequently, several clinical trials are presently investigating the safety and efficacy of gene-modified TolDCs in suppressing autoimmune responses. These trials aim to achieve a more precise understanding of the interactions between gene-modified TolDCs and other inflammatory cells. Furthermore, it should be acknowledged that the utilization of specific gene-modified TolDC may be not suitable for treatment of a variety of autoimmune diseases, including the evaluation of optimal dosages and infusion schedules for genetically-modified TolDC, as well as the determination of appropriate immunosuppressive regimen for a specific disease. Another pivotal facet within the realm of gene-modified TolDC therapy involves the identification of effective and informative assays for monitoring the efficacy and potential adverse immune responses, along with any undesired signs of activation. Ongoing clinical trials, with a focus on precise immune monitoring, are poised to yield the discovery of efficacy biomarkers. These findings will serve as crucial tools for enhancing regulatory cell therapy, with the aim of preventing organ transplant rejection and fostering long-term tolerance. Finally, the incorporation of gene-editing technology is, therefore, the logical next step in advancing TolDC therapy, holding significant implications in the quest for autoimmunity and transplant tolerance.

## Data Availability

No datasets were generated or analysed during the current study.

## Abbreviations

APCs: Antigen-presenting cells
BMDCs: Bone marrow-derived dendritic cells
CREB: cAMP response element binding protein
CIITA: Major histocompatibility complex class II transactivator
DCs: Dendritic cells
EAE: Experimental autoimmune encephalomyelitis
EBI3: Epstein-Barr virus-induced gene 3
GM-CSF: Granulocyte-macrophage colony-stimulating factor
HLA: Human leukocyte antigen
iDCs: Immature dendritic cells
IDo: Indoleamine 2,3-dioxygenase
ILT: Immunoglobulin-like transcript
JAK: Janus kinase
MAPK: Mitogen-activated protein kinases
March1: Membrane-associated ring-CH-type finger 1
mDCs: Mature dendritic cells
MHC: Major histocompatibility complex
MyD88: Myeloid differentiation factor 88
NCoR1: Nuclear receptor corepressor 1
NK: Natural killer
NF-κB: Nuclear transcription factor-kappa B
pDCs: Plasmacytoid dendritic cells
Tregs: Regulatory T cells
SIRT1: Sirtuins 1
SLAP: Src-like adaptor protein
Stat1: Signal transducer and activator of transcription 1
T1D: Type 1 diabetes
TBP-2: Thioredoxin-binding protein-2
TCR: T-cell receptor
Th: T helper
TLR: Toll-like receptor
TNFSF: Tumor necrosis factor superfamily. TolDCs: Tolerogenic dendritic cells
TRAIL: Tumor necrosis factor-related apoptosis-inducing ligand
